# Thymosin β_4_ and β_10_ Expression in Human Organs during Development: A Review

**DOI:** 10.3390/cells13131115

**Published:** 2024-06-27

**Authors:** Gavino Faa, Irene Messana, Pierpaolo Coni, Monica Piras, Giuseppina Pichiri, Marco Piludu, Federica Iavarone, Claudia Desiderio, Giovanni Vento, Chiara Tirone, Barbara Manconi, Alessandra Olianas, Cristina Contini, Tiziana Cabras, Massimo Castagnola

**Affiliations:** 1 Dipartimento di Scienze Mediche e Sanità Pubblica, Università di Cagliari, 09042 Monserrato, Italy; gavinofaa@gmail.com (G.F.); coni@unica.it (P.C.); monipiras@hotmail.com (M.P.); pichiri@unica.it (G.P.); cristinacontini93@unica.it (C.C.); 2 Department of Biology, College of Science and Technology, Temple University, Philadelphia, PA 19122, USA; 3 Istituto di Scienze e Tecnologie Chimiche “Giulio Natta”, Consiglio Nazionale delle Ricerche, 00168 Roma, Italy; imessana53@gmail.com (I.M.); claudia.desiderio@scitec.cnr.it (C.D.); 4 Dipartimento di Scienze Biomediche, Università di Cagliari, 09042 Cagliari, Italy; mpiludu@unica.it; 5 Fondazione Policlinico Universitario A. Gemelli IRCCS, 00168 Roma, Italy; federica.iavarone@unicatt.it; 6 Dipartimento di Scienze Biotecnologiche di Base, Cliniche Intensivologiche e Perioperatorie, Facoltà di Medicina e Chirurgia, Università Cattolica Sacro Cuore, 00168 Roma, Italy; 7 Unità Operativa Complessa di Neonatologia, Fondazione Policlinico Universitario A. Gemelli IRCCS, 00168 Roma, Italy; giovanni.vento@unicatt.it (G.V.); chiara.tirone@policlinicogemelli.it (C.T.); 8 Divisione di Neonatologia, Dipartimento per la Salute della Donna e del Bambino, Università Cattolica del Sacro Cuore, 00168 Roma, Italy; 9 Dipartimento di Scienze della Vita e dell’Ambiente, Sezione Biomedica, Università di Cagliari, 09042 Monserrato, Italy; bmanconi@unica.it (B.M.); olianas@unica.it (A.O.); tcabras@unica.it (T.C.); 10 Laboratorio di Proteomica, Centro Europeo di Ricerca sul Cervello, Fondazione Santa Lucia IRCCS, 00179 Roma, Italy

**Keywords:** human, development, thymosin β_4_, thymosin β_10_, mass spectrometry, preterm newborns, immunostaining

## Abstract

This review summarizes the results of a series of studies performed by our group with the aim to define the expression levels of thymosin β_4_ and thymosin β_10_ over time, starting from fetal development to different ages after birth, in different human organs and tissues. The first section describes the proteomics investigations performed on whole saliva from preterm newborns and gingival crevicular fluid, which revealed to us the importance of these acidic peptides and their multiple functions. These findings inspired us to start an in-depth investigation mainly based on immunochemistry to establish the distribution of thymosin β_4_ and thymosin β_10_ in different organs from adults and fetuses at different ages (after autopsy), and therefore to obtain suggestions on the functions of β-thymosins in health and disease. The functions of β-thymosins emerging from these studies, for instance, those performed during carcinogenesis, add significant details that could help to resolve the nowadays so-called “β-thymosin enigma”, i.e., the potential molecular role played by these two pleiotropic peptides during human development.

## 1. “The Incipit of Our β-Thymosin Adventure”

**Figure 1:**
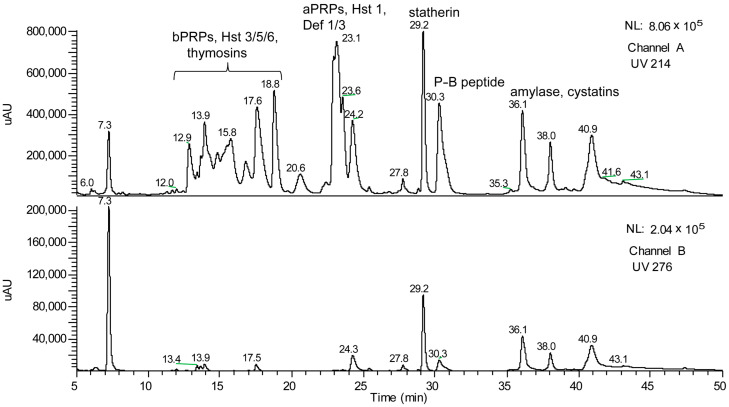
Typical HPLC profile of the acidic soluble fraction of human whole saliva from human adults. The UV profiles revealed by diode-array detection at 214 (**upper lane**) and 276 nm (**bottom lane**) suggests the hypothetical identity of several salivary peptides and proteins based on their polarity and differential UV absorbance [1]. The attributions were confirmed by ESI ion trap and MALDI-TOF mass spectrometry (see Figure 2). (NL: normalization level).

In the early 2000s, our research group carried out the first analysis of the acidic soluble fraction of adult human saliva. At that time, the analytical strategy was mainly based on reversed-phase high-performance-liquid chromatography (RP-HPLC) separation and spectrophotometric diode array detection (DAD) methods [1]. Therefore, the identification and characterization of proteins and peptides belonging to the main families of human salivary proteins could only take profit from two pieces of information: (a) the different detection wavelengths, which accounted for the presence or absence of aromatic amino acid residues (mainly tyrosine and tryptophan) in the structure; and (b) the different polarity of proteins and peptides that affected the retention time in the C18 or C8 reversed-phase columns. For instance, the absence of aromatic amino acid residues in the structure of salivary basic proline-rich proteins (b-PRPs) caused their lack of absorbance at 276 nm, with their elution peaks only detectable at the secondary wavelength of 214 nm. On the contrary, the presence of three tyrosine residues in the statherin sequence allowed us to easily recognize its peak inside the complex and crowded HPLC profile due to its specific absorbance at 276 nm (Figure 1).

**Figure 2:**
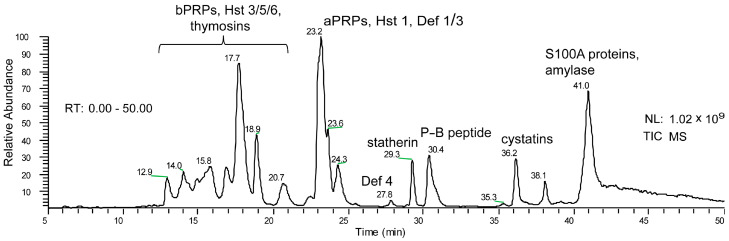
Typical HPLC-TIC (total ion current) profile of the acidic soluble fraction of human whole saliva from human adults obtained by revealing, with IT-MS, the ions generated from eluting analytes at the electrospray (ESI) source. The TIC profile contained ESI spectra useful to determine the M (average) of the most abundant components of the main families of human salivary proteins [7].

The easy and painless collection of the samples encouraged us to continue investigating human saliva with the ambitious aim of characterizing its proteome. However, the need to exploit a detection method capable of providing useful information for the unambiguous characterization of the multiple salivary components was immediately clear. The newly available ion-trap (IT) mass spectrometers (MSs) coupled with HPLC via electrospray ionization (ESI) sources were helpful for our purposes; so, we initially utilized a low-resolution IT-MS (Deca-XP, Thermofisher, Waltham, MA, USA), which allowed us to characterize many components of the human adult salivary proteome [2,3,4,5,6] (Figure 2).

**Figure 3:**
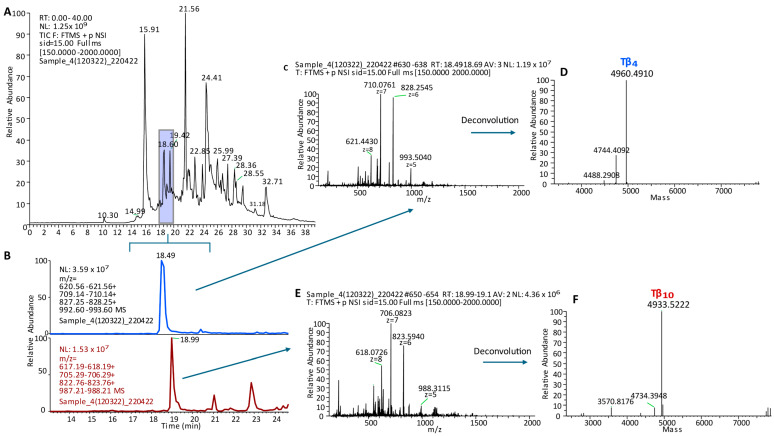
Panel (**A**): High-resolution (Orbitrap) HPLC-ESI-MS profile of the acidic soluble fraction of whole saliva from preterm newborns (233 days of post-conceptional age (PCA)). The elution range (18–20 min) where Tβ_4_ and Tβ_10_ elute is evidenced by the colored box. Panel (**B**): XIC (eXtracted Ion Current) peaks were revealed by selecting four multicharged ions from [M + 8H^+^]^8+^ to [M + 5H^+^]^5+^ of Tβ_4_ (**upper lane**) and Tβ_10_ (**bottom lane**). The ESI spectrum and the corresponding deconvolution of Tβ_4_ are shown in Panels (**C**,**D**), respectively. The ESI spectrum and the corresponding deconvolution of Tβ_10_ are shown in Panels (**E**,**F**), respectively. Deconvolutions allowed us to determine the experimental M (monoisotopic) of Tβ_4_ (Panel (**D**), theor. 4960.486) and Tβ_10_ (Panel (**F**), theor. 4933.523).

**Figure 4:**
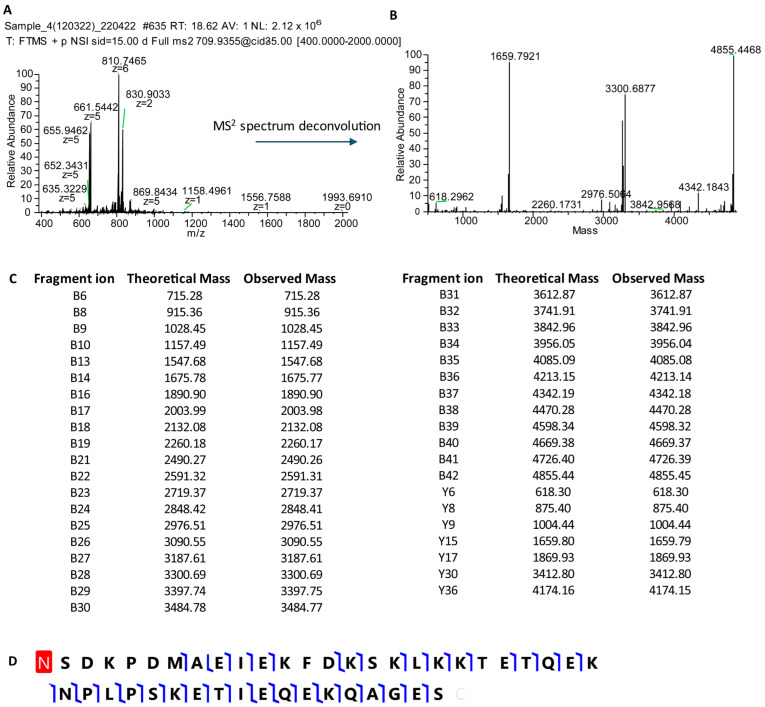
MS/MS characterization of the sequence of Tβ_4_. High-resolution MS/MS of the [M + 7H^+^]^7+^ ion at 709.94 *m*/*z* of Tβ_4_ (Panel (**A**)) and the corresponding deconvoluted spectrum (Panel (**B**)). Matching fragments’ grid (Panel (**C**)), showing theoretical and experimental B and Y fragment ions (blu lines) obtained by ProSight Lite (version 1.4), assuming the N-term acetylation of Tβ_4_ (red box) as reported in the graphical fragment map (Panel (**D**)).

**Figure 5:**
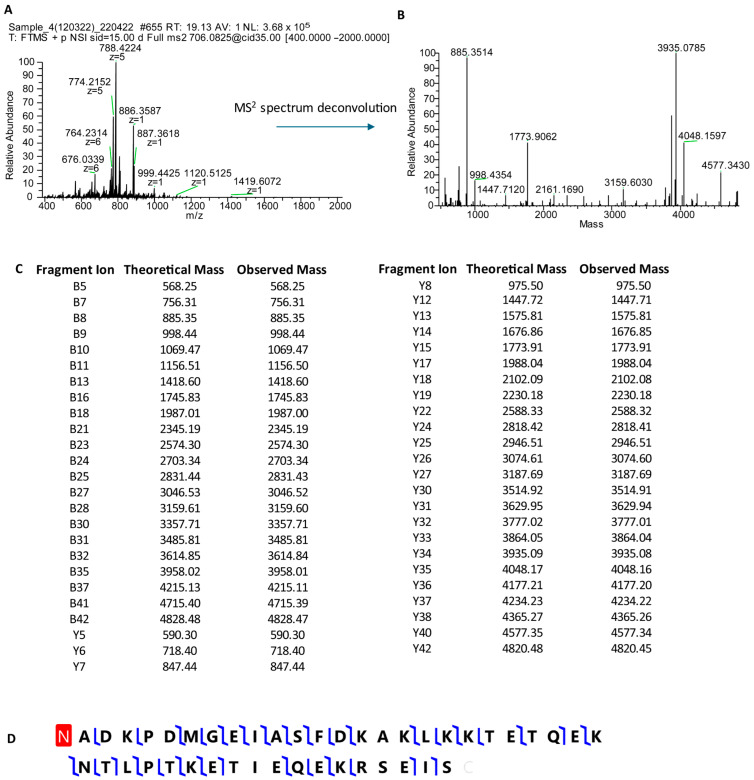
MS/MS characterization of the sequence of Tβ_10_. High-resolution MS/MS of the [M + 7H^+^]^7+^ ion at 706.08 *m*/*z* of Tβ_10_ (Panel (**A**)) and the corresponding deconvoluted spectrum (Panel (**B**)). Matching fragments’ grid (Panel (**C**)), showing theoretical and experimental B and Y fragment ions (Blu lines) obtained by ProSightLite (Version 1.4), assuming the N-term acetylation of Tβ_10_ (red box), as reported in the graphical fragment map (Panel (**D**)).

Unlike the proteomic studies carried out by other groups, our analytical strategy did not involve a proteolytic treatment of the samples. Instead, our goal was to characterize intact small proteins and peptides soluble in the supernatant of acidic solutions obtained by diluting (1:1 *v*/*v*) whole salivary samples either with trifluoroacetic acid (TFA 0.2%) or formic acid (FA 0.2%). This pipeline was lately defined top-down proteomics [7,8], and it is still utilized by us and other groups. Since one of the most intriguing issues of the proteomic analysis of a bodily fluid, such as saliva, is the comprehension of the contribution of the different sources to the whole, around the year 2004, we collected and analyzed the gingival crevicular fluid (GCF). GCF is a physiological fluid arising from the gingival plexus of blood vessels in the gingival corium, subjacent to the epithelium lining the dental–gingival space. It contains a diverse population of cells, including bacteria from the adjacent plaque mass, transmigrating leucocytes, and desquamated epithelial cells, which are passively washed out into the oral cavity [9]. The HPLC analysis of GCF coupled with DAD and ESI ion trap MS detection led us to recognize different peptides and proteins soluble in acidic solution [10]. Besides high quantities of human serum albumin, a-defensins 1–4, and minor amounts of cystatin A, P-B peptide, and statherin, two peptides with the average masses (Mav) of 4964.0 ± 0.6 Da and 4936.4 ± 0.6 Da were detected. At that time, we were unable to characterize their amino acid sequence due to the low resolution of the MS/MS fragmentation spectra. A few months later, we hypothesized that the two peptides detected in GCF were thymosin beta 4 (Tβ_4_, Mav theor 4963.5 Da, Swiss-Prot code P62328) and thymosin beta 10 (Tβ_10_, Mav theor 4936.5 Da, Swiss-Prot code P63313), because of the information published in the papers of Prof. Allan L. Goldstein [11,12], Emeritus at the Department of Biochemistry and Molecular Biology of George Washington University (Washington, DC, USA) and discoverer of thymosins, a family of at least 40 mostly small acidic polypeptides, with molecular weights ranging from 1000 to 15,000 Da. During that period, our research group, aided by the availability of the new-generation Orbitrap analyzers, started a study devoted to the characterization of the proteome of whole saliva collected from preterm newborns in comparison with the salivary proteome of infants of different ages and adults [13]. Among the striking differences with respect to adults, the analysis of the preterm newborns’ salivary proteome evidenced considerably high levels of Tβ_4_ and Tβ_10_ and allowed the unambiguous determination of their amino acid sequences with an N-terminal acetylation by high-resolution MS/MS fragmentation (Figure 3, Figure 4 and Figure 5) [12].

The mean concentration of Tβ_4_ (measured by HPLC-ESI-MS using β-thymosin standards as references) in the saliva of preterm newborns at approximately 200 days of post-conceptional age (PCA) was about 2.0 nanomol/mL, 20 times higher than the mean concentration measured in adults [13]. Indeed, Tβ_4_ was firstly identified in human adult saliva using an enzymatic immunoassay, always by the group of Prof. Goldstein [14,15], at a concentration ranging from about 0.2 up to 2 µg/mL (approximately from 0.04 to 0.40 nanomol/mL). In the selected glandular saliva, the concentrations of Tβ_4_ and Tβ_10_ had to be lower than the detection limit of our analytical technique. However, they were undetectable; so, we assumed that the main contribution of Tβ_4_ and Tβ_10_ to human saliva derived from GCF [16]. In the saliva of preterm newborns, the concentration of Tβ_10_ (≈0.5 nanomol/L) was roughly in a constant ratio of ≈1:4 with respect to Tβ_4_ [13].

## 2. Structural Characteristics of Human Thymosin Beta 4 and Thymosin Beta 10

**Figure 6:**
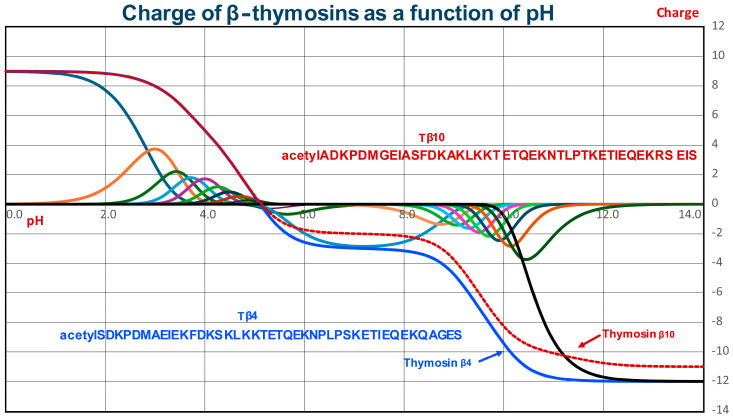
Charge of β-thymosins as a function of pH (blue line, Tβ_4_; dashed red line, Tβ_10_). The curves were obtained as reported in [17]. The two lines resulted from the summation of the molar fractions of the 22 charged species multiplied for their charge (from +9 to −12, reported with different colors only for Tβ_4_). For simplicity, the molar fractions of the 21 charged species of Tβ_10_ (from +9 to −11) are not shown.

As shown in Figure 4 and Figure 5, Tβ_4_ is a small and very polar peptide of 43 amino acids, 21 of which are ionogenic, i.e., 3 Asp, 8 Glu, 9 Lys, and the C-terminal Ser, while the N-terminus is acetylated. The prevalence of acidic versus basic residues generates a pI close to five and a net charge close to −3 at physiological pH (Figure 6) [17].

Tβ_10_ has one aspartic acid residue less than Tβ_4_; thus, its charge at physiological pH is about −2. These features result in highly reactive and flexible structures, typical of disordered proteins. Indeed, β-thymosins are included in the family of intrinsically disordered proteins, defined as unstructured proteins in their free conformation but able to adopt a well-defined three-dimensional structure when interacting with binding partners. Moreover, both Tβ_4_ and Tβ_10_ undergo disorder-to-order transitions in carrying out their biological functions [18].

A common structural attribute of β-thymosins is the presence of one or more βT repeats, an approximate 43 amino acid signature motif first defined for the prototypical Tβ_4_. Tβ_4_ was discovered in the 1970s during the purification of polar peptides from the thymus tissue, hence the name [11]. Thereafter, various investigations showed that Tβ_4_ binds and sequesters monomeric G-actin, the major component of the cytoskeleton in eukaryotic cells [19]. Nuclear magnetic resonance (NMR) structural studies on isolated Tβ_4_ began and subsequently on the Tβ_4_-actin complex [20,21]. X-ray crystallography studies were only possible when procedures to stabilize the intrinsically disordered Tβ_4_ in its complex with actin were developed [22,23]. These structural data, together with the discovery of homologs through protein and nucleotide sequencing, allowed a precise definition of the βT repeat characteristics: a consensus LKKT motif at the center of the sequence, flanked by two somewhat conserved segments that weakly form amphipathic α-helices in isolation, stabilizing when bound to actin. Because the LKKT motifs, N-terminal α-helices, and actin-binding properties were detected in the Wiskott–Aldrich syndrome protein (WASP) homology domain 2 (WH2)-containing proteins, βTs and WH2s domains are often grouped in the same functional superfamily [24,25].

βT repeats appear within proteins either singly or as multiple repeats. Tβ_4_ and the close homologs, such as Tβ_10_, contain one βT repeat, while other members contain from 2 to 27 such repeats [26]. One widely investigated multi-βT repeat protein is the three βT repeat Drosophila ciboulot, which was the first protein having an actin-bound βT repeat structure investigated by X-ray crystallography [27]. Single- and multi-repeat βTs have differences in their actin-binding functionality: all the studied single-repeat βTs act like Tβ_4_ in inhibiting their bound actin from forming or joining a filament [28].

Around the year 2007, the antibodies for the immunohistochemical analyses of both β-thymosins were commercially available; so, we decided to investigate by immunohistochemistry the expression and distribution of Tβ_4_ and Tβ_10_ in various tissues of fetuses, infants, children, and adults at autopsy. As a positive control tissue, we used a biopsy of skeletal muscle, in which the antibody against Tβ_4_ immunostained many muscle cells. The results of our studies are reviewed in the following sections.

## 3. Salivary Glands

**Figure 7:**
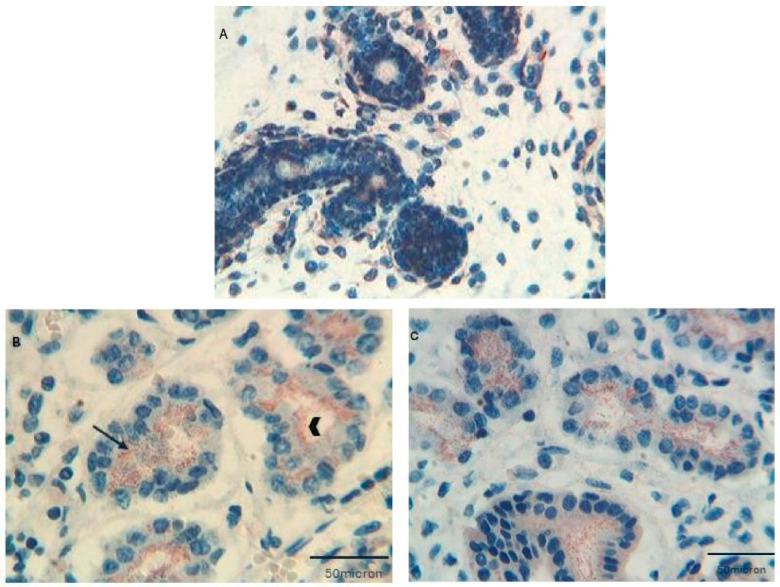
Immunostaining for Tβ4 in salivary glands. (**A**) Immunostaining for Tβ4 in fetal minor salivary glands shows granular reactivity for the peptide in tubular cells and in the surrounding myxoid stroma (Magnification ×100). (**B**) Granular immunoreactivity for Tβ4 in the cytoplasm of developing tubular cells in submandibular fetal glands. The peptide is also expressed in the lumen (arrowhead) of developing glands. (Magnification ×250). (**C**) Fetal parotid gland showing immunostaining for Tβ4 in tubular cells undergoing branching morphogenesis. (Magnification ×250).

As a starting point, we analyzed the expression of both Tβ_4_ and Tβ_10_ in the parotid, submandibular, and minor salivary glands localized at the base of the tongue in human fetuses and preterm newborns, ranging in PCA from 12 to 31 weeks and in a 1.5-year-old child [13]. In this study, a high expression of Tβ_4_ was observed in major and minor developing salivary glands at all gestational ages, with peculiar features regarding the expression of the peptide at different stages of development. At 12 and 13 weeks of intrauterine life, Tβ_4_ was mainly expressed as granular deposits inside the cytoplasm of tubular cells and inside the lumen of salivary gland tubules (Figure 7A).

Tβ_4_-reactive granules were also detected in the cytoplasm of acinar cells of the developing acinar structures (Figure 7B,C).

At this PCA, Tβ_4_ was also highly expressed in the stroma surrounding the developing epithelial structures, inside the cytoplasm of activated fibroblast precursors embedded in the mesenchyme of the fetal glands. Marked differences were observed at 31 weeks of gestation. Contrasting with the granular expression in tubular and acinar cells observed at the previous ages, immunoreactivity for Tβ_4_ appeared to be widespread to the whole cytoplasm of acinar and tubular cells, suggesting the binding of Tβ_4_ to the actin of the cytoskeleton. Few small Tβ_4_-reactive granules were restricted to the lumen of tubular structures. A decrease in Tβ_4_ expression was noticed, at this gestational age, in the fibroblasts of the glandular mesenchyme. Changes were also observed in the salivary glands at 1.5 years of postnatal age, in which immunoreactivity for Tβ_4_ was characterized by a mild and diffuse immunostaining in the cytoplasm of tubular cells. A mild granular staining was observed in the cytoplasm of some acinar cells. No reactivity was found in the salivary gland stroma at this age. Moreover, in the salivary glands of the adult subjects utilized as a control group, a mild diffuse Tβ_4_ expression was restricted to the cytoplasm of tubular cells, in the absence of any significant reactivity in acinar cells and in the surrounding stroma. These data, taken together, clearly indicate that the expression of Tβ_4_ in the human salivary glands is dynamic, changing significantly during the different stages of prenatal and postnatal development. The predominant granular pattern found in the early stages of development indicates a prevalent localization of the peptide inside cytoplasmic vesicles, suggesting a secretory pathway that was confirmed by the finding of Tβ_4_ granules in the lumen of salivary gland tubules. This finding was in line with the presence of very high levels of Tβ_4_ secreted into the saliva of preterm newborns as compared to adults [13]. In the 1.5-year-old child, the prevalent granular pattern of Tβ_4_ immunostaining appeared progressively substituted by a diffuse reactivity in the cytoplasm of tubular cells, with small, rare granules in acinar cells. This result suggests a switch in Tβ_4_ function, changing from a secretory immunophenotype to a structural phenotype, in which Tβ_4_ may be associated with other cytoplasmic components, including actin of the cytoskeleton. In adults, a weak diffuse immunoreactivity for Tβ_4_ restricted to tubular cells was detected.

Another interesting finding, which arose from our immunohistochemical studies, was concerned with the Tβ_4_ expression in the stroma of the salivary glands, with large granular deposits localized in the cytoplasm of fibroblast precursors, embedded in the glandular microenvironment (GME), surrounding the developing tubular and acinar structures in the early stages of development. This result may suggest a major role for the salivary gland-associated fibroblasts in the branching morphogenesis of human salivary glands and in acinar differentiation. In recent years, the role of fibroblasts during embryonal development has been underlined by multiple studies, revealing their abilities in the production of complex extracellular matrix, in providing niches and positional information for stem/progenitor cells and in driving morphogenesis [29,30]. The evidence of Tβ_4_ production by the fibroblasts of the fetal salivary glands reinforces the hypothesis of a key role of fibroblasts in human development, suggesting that the Tβ_4_ produced and secreted by these cells might influence the branching morphogenesis of the epithelial structures and the development of salivary glands. Accepting the hypothesis that Tβ_4_ might play a major role during development as confirmed by our findings, and previously reported by the decreased Tβ_4_ levels in the saliva after birth, in childhood, and in adulthood [13], a secretion switch should be expected after birth.

**Figure 8:**
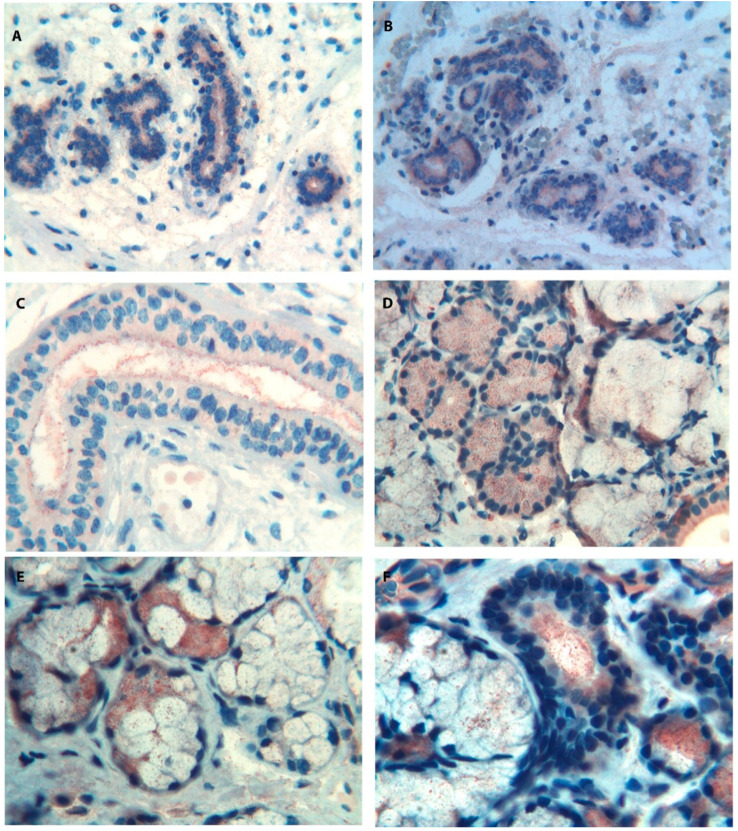
Immunostaining for Tβ_10_ in salivary glands. (**A**) Tβ_10_ expression in the developing fetal parotid gland is characterized by a granular immunostaining in the cytoplasm of immature tubular cells undergoing branching morphogenesis. A mild reactivity is also observed in the myxoid stroma. (magnification ×100). (**B**) Immunoreactivity for Tβ_10_ in the developing salivary glands is mainly localized in the cytoplasm and in the lumen of tubular cells. A mild reactivity is detected in the surrounding stroma. (magnification ×100). (**C**) A large tubular structure of a fetal salivary gland shows abundant granular deposits of Tβ_10_ inside the lumen. The peptide is also expressed, at low levels, in the cytoplasm of tubular cells. (magnification ×250). (**D**) Immunoreactivity for Tβ_10_ in minor salivary glands in Sjögren syndrome is characterized by a granular cytoplasmic pattern mainly localized in serous cells. A mild diffuse cytoplasmic reactivity is also found in the cytoplasm of mucous cells (magnification ×100). (**E**) In serous–mucous acinar structures, immunoreactivity for Tβ_10_ is predominantly found in serous cells with scattered small Tβ_10_ granules in mucous cells and in the peri-glandular stroma. (magnification ×400). (**F**) Tβ_10_ is detected in the lumen of salivary gland tubular structures. The peptide is also found in the cytoplasm of some mucous cells. (magnification ×400).

A peculiar pattern of expression (different from the pattern described for Tβ_4_) was found for Tβ_10_ in developing salivary glands [31]. Immunoreactivity for Tβ_10_ was analyzed in the major and minor salivary glands in two human fetuses and four preterm newborns, ranging from 13 up to 33 weeks of PCA. Tβ_10_ immunoreactivity was detected in all the examined salivary glands. Moreover, the parotid glands showed the highest Tβ_10_ reactivity, characterized by granular staining in the cytoplasm of tubular cells undergoing branching morphogenesis (Figure 8A).

In the developing glands, immunoreactivity for Tβ_10_ was also observed in the surrounding myxoid stroma (Figure 8B).

Lower levels of immunoreactivity for Tβ_10_ were observed in minor salivary glands. Marked changes were observed in Tβ_10_ expression and localization during embryogenesis. Tβ_10_ was mainly localized extracellularly in the cytoplasm of fibroblast-like cells embedded in the stroma surrounding the epithelial structures, in the youngest human fetuses (13 weeks of PCA), in the cytoplasm of immature duct cells at 20 weeks, and in acinar cells and in the duct lumen at 33 weeks of PCA (Figure 8C).

The strong expression of Tβ_10_ in the human salivary glands during the initial phases of the development suggested a major role for the peptide in the organogenesis of salivary glands. The preferential localization of the peptide in the salivary gland-associated fibroblasts laid stress on the role of this cell type during organogenesis and, particularly, in the branching morphogenesis of salivary glands, paralleling the role previously hypothesized for Tβ_4_.

Marked differences regarding the role and the expression of Tβ_4_ and Tβ_10_ have been found even in the pathology of salivary glands, in patients affected by Sjӧgren syndrome [32]. At immunohistochemistry, in patients with primary Sjӧgren syndrome, minor salivary glands showed a peculiar pattern characterized by immunoreactivity for Tβ_10_ in acinar cells in the absence of any immunostaining for Tβ_4_. Immunostaining for Tβ_10_ was mainly detected in serous cells, with a granular pattern diffuse to the entire cytoplasm (Figure 8D).

A mild diffuse reactivity for the peptide was observed in the cytoplasm of ductal cells. In glands with a mixed serous–mucous component, immunoreactivity for Tβ_10_ allowed the identification of serous cells (Figure 8E).

A mild granular reactivity was detected in the peri-glandular stroma. Tβ_10_ was also found in the lumen of salivary gland ducts, as well in the cytoplasm of some mucous cells (Figure 8F).

All these results suggested a different role for Tβ_4_ and Tβ_10_ in patients with primary Sïogren syndrome. The immunohistochemical findings correlated with differences in beta thymosin expression in saliva of the two peptides: Tβ_10_ was detectable in 66.7% of patients with primary Sjӧgren syndrome, while Tβ_4_ sulfoxide was detectable only in 44.4% of patients.

## 4. Gut, Liver, and Pancreas

The finding of high levels of Tβ_4_ in the saliva, the body fluid that is continuously ingested, induced us to analyze the expression of the peptide in the human gut during development and in adulthood. To this aim, the expression of Tβ_4_ was analyzed by immunohistochemistry in autoptic samples from two fetuses of 20 and 21 weeks of PCA and two 65- and 74-year-old adults. Marked differences were observed between the adult subjects and the fetuses. In the fetal esophagus, a thin layer expressing Tβ_4_ was detected on the surface of the epithelium, whereas the adult esophagus showed a mild reactivity for the peptide in the cytoplasm of the epithelial cells of the superficial layers. In the fetal stomach, Tβ_4_-immunoreactive granules were observed in many columnar cells, suggesting their ability to secrete the peptide during gestation. On the contrary, in the adult stomach, the gastric mucosa appeared covered by a superficial Tβ_4_-reactive thin layer. A mild reactivity for Tβ_4_ was also found in the chief and oxyntic cells in the gastric glands. In the fetal ileum, Tβ_4_-reactive granules were observed in the mucous cells of the intestinal villi and in the mucus occupying the intestinal lumen. In the adult ileum, two patterns of Tβ_4_ reactivity were identified: diffuse and homogeneous in the cytoplasm of enterocytes covering the villi and granular in the cytoplasm mucous cells of the villi. These findings suggested the existence of striking differences regarding the function of Tβ_4_ in different cell types: a secretory (granular expression) phenotype in mucous cells and a structural (homogeneous immunostaining) phenotype, putatively reflecting the adhesion of Tβ_4_ to actin of the cytoskeleton in the enterocytes [33]. Fetal and adult Tβ_4_ patterns were more similar in the mucosa of colon and rectum. In fetal colonic mucosa, coarse granules were found in superficial epithelial cells, whereas, in adult colon mucosa, a diffuse reactivity, with coarse perinuclear granules, suggesting a localization of the peptide in the trans-Golgi network, was detected in superficial and crypt epithelial cells.

Striking differences regarding Tβ_4_ expression were found between the fetal and the adult livers. In the fetal liver, Tβ_4_ was not expressed in the hepatocytes, in bile duct cells, or in the developing ductal plate. A strong Tβ_4_ immunostaining was restricted to undifferentiated stem/progenitor cells localized in the immature portal tracts. In the adult liver, the expression pattern changed completely, with a strong granular immunoreactivity in the cytoplasm of hepatocytes and in Kupffer cells in the absence of any significant reactivity in bile duct cells [34]. Intriguing data were reported in a study on Tβ_4_ and Tβ_10_ expression in hepatocellular carcinoma (HCC). Tβ_4_ immunostaining was found at low levels in 30% of cases, being negative in the vast majority of HCCs. On the contrary, Tβ_10_ reactivity was detected in 97% of HCCs [35]. The higher incidence of Tβ_10_ was paralleled by its strong expression in tumor cells involved in stromal invasion, suggesting a major role for Tβ_10_ in liver carcinogenesis and in HCC progression. Our results were confirmed later by other groups, which underlined the utility in clinical practice of immunostaining for Tβ_10_ expression as a predictor of poor prognosis in HCC [36].

Nevertheless, the findings regarding Tβ_4_ expression in the liver deserve some consideration. Whereas our preliminary data were in favor of a prevalent Tβ_4_ expression in human organs and tissues during fetal life, followed by a decrease after birth, the liver pattern forced us to modify this hypothesis. In liver cells, Tβ_4_ expression was characterized by low Tβ_4_ levels during gestation, high levels in the postnatal life and in adulthood, and low expression levels in cancer. This means that the dynamics of Tβ_4_ expression in different cell types are more complex than previously thought, and caution should be taken in the evaluation of the function of this peptide in every cell and tissue. One hypothesis is confirmed by our findings regarding Tβ_4_ expression in liver cells: a similarity regarding the Tβ_4_ expression pattern between fetuses and cancer originating from the same cell type, which puts Tβ_4_ at a crossroads between development and cancer [37]. Regarding the hepatic expression pattern, the low levels in the fetal liver were paralleled by low levels in HCC.

**Figure 9:**
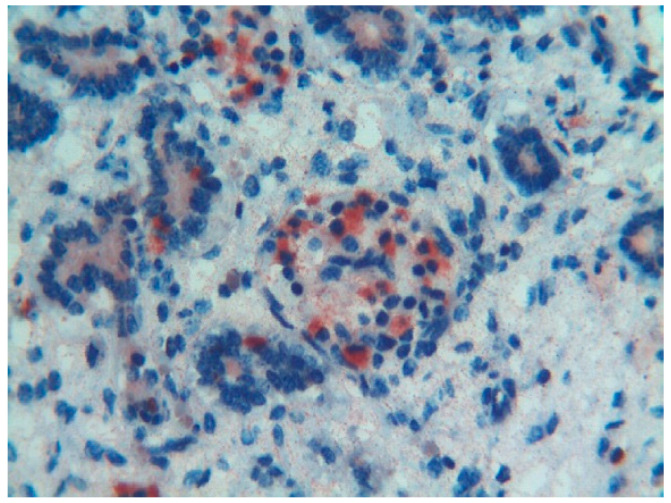
In the fetal pancreas, Tβ_10_ is mainly expressed in the endocrine cells of the developing Langerhans islets, showing a strong homogeneous cytoplasmic immunostaining. A mild granular reactivity for Tβ_10_ is also observed in the developing tubular structures of the cells of the exocrine pancreas and in the stroma of the pancreas microenvironment. (magnification ×100).

In the fetal developing pancreas, immunostaining for Tβ_10_ was mainly found in the endocrine pancreas, inside the developing Langerhans islet cells. Tβ_10_ was localized in the cytoplasm of a subset of endocrine cells, showing a diffuse homogeneous cytoplasmic reactivity (Figure 9).

In addition, a mild granular immunoreactivity for Tβ_10_ was detected in the developing exocrine pancreas, inside the cytoplasm of tubular cells undergoing branching morphogenesis (Figure 9). Tβ_10_ was also expressed in the adult pancreas. Scattered Tβ_10_ positive granular deposits were also observed in the myxoid stroma of the fetal pancreas [38].

Taken together, these findings indicated the existence of a cell-specific and a differentiation stage-specific regulation of the expression pattern of Tβ_4_ and Tβ_10_ in the gut and in the annexed glands during development and in adulthood. The marked changes observed regarding the immunohistochemical patterns observed in different tracts of the gastrointestinal (GI) tract, as well in the liver and pancreas, probably reflected the different roles played by this peptide during the organogenesis and the changing functions of Tβ_4_ in childhood and in adult life. The granular immunoreactivity indicated the localization of the peptide in the cytoplasmic vesicles, suggesting a secretory phenotype. The larger Tβ_4_-reactive granular deposits in the perinuclear site might indicate a localization of the peptide in the trans-Golgi apparatus, showing the localization of the peptide in the secretory pathway. The homogenous diffuse cytoplasmic staining could indicate the association of Tβ_4_ with the actin of the cytoskeleton, suggesting a structural actin-monomer sequestering function assumed by Tβ_4_ in cells. New intriguing findings regarded the strong expression of the hepatocytes in the adult liver, which contrasted with the absence of reactivity for Tβ_4_ in the fetal hepatocytes. These findings confirmed the existence of organ-specific patterns regarding the different functions of Tβ_4_ in the development of different human organs and tissues. High levels in the saliva from preterm newborns and in the salivary glands probably reflected a major role for the peptide in their development and contrasted with the low levels of expression in the developing liver, probably suggesting a minor role for Tβ_4_ in hepatic development. On the other hand, the diffuse granular reactivity in adult hepatocytes suggested a major role for the liver in the production and secretion of Tβ_4_ in adult life. The high levels of Tβ_4_ detected in the fetal GI tract stress the key role played by Tβ_4_ in the organogenesis of the human GI tract. The heterogeneity of Tβ_4_ immunoreactivity in the different regions of the GI tract, ranging from the absence in the esophageal mucosa to the high and diffuse expression in the colon mucosa, associated with the marked differences between prenatal and adult life, should be considered when evaluating the role of Tβ_4_ in human development and physiology.

## 5. Genitourinary Tract

**Figure 10:**
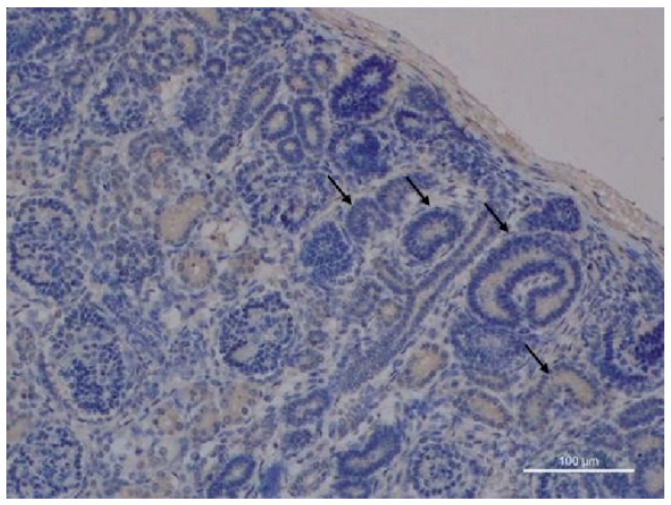
Fetal kidney, cortical zone: a mild diffuse immunostaining for Tβ_4_ is observed in Comma-shaped and S-shaped bodies (arrows). No reactivity for the peptide is found in the collecting tubules and in glomeruli. (magnification ×50).

The first studies on the expression of Tβ_4_ in the fetal and adult genitourinary tract, including kidneys, bladder, uterus, ovaries, testicles, and prostate, revealed the expression of the peptide in cells of different origin, such as surface epithelial cells, gland cells, and interstitial cells in all the organs analyzed, with marked differences, in some cases, between fetal and adult organs. The initial analyses of Tβ_4_ in fetal kidneys evidenced a similar pattern of expression between fetal and adult organs, characterized by a weak and homogeneous cytoplasmic immunostaining for Tβ_4_ in the primary and secondary ducts. A weak immunostaining for Tβ_4_ was detected in S-shaped and Comma-shaped bodies in the cortex of the developing kidney in the absence of any significant reactivity in glomeruli (Figure 10).

In the fetal bladder, the transitional epithelium showed a homogeneous weak reactivity for Tβ_4_; a similar diffuse expression of Tβ_4_ was also found in the underlying stromal cells. Conversely, in the adult bladder, immunostaining in the transitional epithelium was granular, often characterized by coarse perinuclear deposits suggesting a localization of the peptide in the trans-Golgi apparatus. A focal reactivity for the peptide was also found in stromal cells. Marked differences were found in the endometrium between fetuses and adult women. No reactivity for Tβ_4_ was found in the fetal developing endometrial glands, whereas focal immunostaining for the peptide was observed in scattered stromal cells. In the adult endometrium, granular cytoplasmic immunoreactivity was detected in the endometrial glands, in absence of any reactivity in stromal cells. No immunostaining for Tβ_4_ was revealed in the fetal prostate, both in the glands and in stromal cells, contrasting with a diffuse reactivity for Tβ_4_ restricted to the stroma of adult subjects. In the fetal testicle, no expression of Tβ_4_ was found in the immature spermatic ducts, whereas some mild expression of the peptide was found in scattered interstitial cells. The pattern of Tβ_4_ expression changed in the adult testicle, with a granular expression of Tβ_4_ in the interstitial cells associated with a weak immunostaining in the cells of the spermatic ducts. A similar pattern was found in fetal and adult ovaries. The peptide was mainly expressed in the cytoplasm of oocytes, appearing as a fine granular reactivity in fetal organs and as a homogeneous weak reactivity in adult organs. A diffuse immunoreactivity for Tβ_4_ was also observed in stromal cells, both in fetal and adult ovaries, confirming the key role of the stromal component in the development of fetal organs [39].

This study demonstrated that Tβ_4_ plays a major role during human development, even in the genitourinary tract, being expressed in all organs analyzed, and confirmed that Tβ_4_ expression may change significantly during postnatal and adult life. Moreover, these findings demonstrated that Tβ_4_ expression is specific for each different organ, and it appears to be restricted to certain structures or to specific cell types. Furthermore, the type of reactivity may change significantly with age within the same cell, being granular in fetal oocytes and homogeneous in adult oocytes. These changes related to the Tβ_4_ pattern of expression might indicate a different role of the peptide at different ages: secretory, concentrated in cytoplasmic vesicles in the fetal age, and structural, homogeneously associated with the cytoskeleton components in adult life. The evidence of relevant Tβ_4_ expression in multiple adult organs, including kidneys, clearly indicates that the functions of Tβ_4_ are not restricted to the prenatal life and to human development and might be important even in adult life. Regarding the different cell types expressing Tβ4, the finding of immunostaining for Tβ_4_ in the stromal cells of multiple organs, including ovaries and testicles, indicated that Tβ_4_ functions are not restricted to epithelial cells, but may include stromal cells and fibroblasts as previously found in the development of salivary glands [13]. Finally, the high expression of Tβ_4_ in fetal oocytes and in adult oocytes suggested a role for this peptide both in germ cell development and in its maintenance in adult women.

## 6. Thymosin β_4_ and Thymosin β_10_ in Human Nephrogenesis

**Figure 11:**
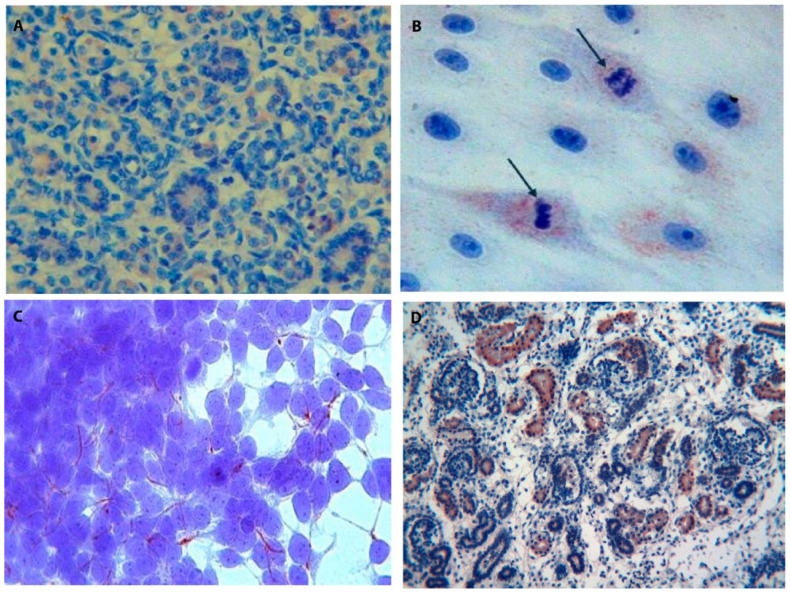
Tβ_4_ and Tβ_10_ in human nephrogenesis. (**A**) Fetal kidney medullary zone: immunoreactivity for Tβ_4_ is shown in the cytoplasm of tubular cells. (magnification ×100) (**B**) PK1 renal cell line. Scattered cultured renal cells immunostained for Tβ_4_ show a vesicular cytoplasmic staining, which appears stronger in mitotic cells (arrows) (magnification ×630). (**C**) 293T cell line. Embryonic renal progenitor cells show a peculiar expression pattern for Tβ_4_. The peptide is mainly expressed in thin cytoplasmic processes, suggesting a role for Tβ_4_ in intercellular communications. (magnification ×630). (**D**) Immunostaining for Tβ_10_ in the fetal kidney is mainly restricted to the proximal and distal tubules. No reactivity for the peptide is observed, in this picture, in the glomerular cells. (magnification ×100).

The preliminary findings on Tβ_4_ expression in the fetal kidney induced us to better analyze Tβ_4_ and Tβ_10_ expressions in a larger series of fetuses and newborns, ranging from 17 up to 38 weeks of gestation, to highlight their role in the different phases of the intrauterine life. These further investigations allowed us to reach a better picture of Tβ_4_ expression in the various components of the developing human kidney and in the multiple cell types involved in nephrogenesis [40]. The analysis of all fetal kidney samples immunostained for Tβ_4_ first confirmed that Tβ_4_ is scarcely expressed in the stem/progenitor cells of the subcapsular nephrogenic zone and in the epithelial components of the cortex, including glomeruli, proximal, distal, and collecting tubules (Figure 10). A focal cytoplasmic reactivity was detected, only in few cases, in distal tubules and in the medullary zone, restricted to the Henle loops (Figure 11A).

The highest levels of immunoreactivity for Tβ_4_ were found in the stromal cells localized in the renal capsule and in the hilum surrounding the ureter and the branches of the renal artery. A strong reactivity for the peptide was also found in the smooth muscle cells of the arterial wall and in the perineural cells encircling nerves. Tβ_4_-reactive fibroblast-like cells were also found encircling the Bowman capsule and distal tubules in the absence of any staining in stromal cells surrounding proximal tubules [41]. When immunoreactivity for Tβ_4_ was analyzed in renal cells in culture, the peptide was expressed in the cytoplasm, showing a vesicular pattern of immunostaining. Cells in mitosis were characterized by a strong expression of Tβ_4_ compared to adjacent cells (Figure 11B).

A different pattern of expression was observed in the 293T cell line, adenovirus-immortalized human embryonic cells, in which Tβ_4_ was mainly expressed in cytoplasmic projections, a pattern suggestive of a role of Tβ_4_ in intercellular communications (Figure 11C).

A diverse pattern of expression was observed regarding Tβ_10_ in the developing human kidney of human fetuses and preterm infants, ranging from 25 up to 36 weeks of gestation [42]. Tβ_10_ was highly expressed in the proximal and distal tubules, whose cells were characterized by a strong homogeneous cytoplasmic reactivity for the peptide (Figure 11D).

No immunostaining was found in the collecting ducts and in the glomeruli. Focal immunostaining for Tβ_10_ was detected inside rare glomeruli in some fetuses with gestational age lower than 29 weeks of gestation. Unlike Tβ_4_, Tβ_10_ was also detected in the nephrogenic zone located in the subcapsular region occupied by the nephrogenic mesenchymal stem/progenitor cells. In this area, Tβ_10_ showed granular immunoreactivity, mainly identified in the cytoplasm of the Comma-shaped and S-shaped bodies. Interestingly, Tβ_10_ expression disappeared in the S-shaped bodies with the appearance of the primitive vascular tuft followed by the generation of the glomerular body. In the adult kidney, immunostaining for Tβ_10_ was restricted to the proximal and distal tubules in the absence of any expression inside glomeruli.

These findings first confirmed the selective localization of beta-thymosins in developing organs, including the fetal kidney, and evidenced once more of their different expression in the fetal and adult kidneys. Striking differences should also be underlined between Tβ_4_ and Tβ_10_ expression. The most intriguing finding regarding Tβ_10_ expression was represented by its reactivity in the nephrogenic zone, participating in the early phases of nephrogenesis and disappearing at the early insurgence of the glomerular tuft. This finding contrasted with the absence of any significant reactivity for Tβ_4_ in the mesenchymal progenitors, highlighting the hypothesis of different functions of the two β-thymosins during development. Another difference regarding immunostaining for Tβ_4_ and Tβ_10_ was the restriction of the reactivity for the former in the cytoplasm, whereas Tβ_10_ was also localized inside the nuclei of tubular cells.

## 7. Thymosin β_4_ Expression in Mast Cells of Fetal Tissues and in the Tumor Microenvironment

To test immunoreactivity for Tβ_4_ in the different subtypes of mast cells, the expression of the peptide was analyzed in multiple fetal tissues, in colon biopsies containing mucosal mast cells, in skin biopsies with dermal-serosal mast cells, and in biopsies from salivary gland tumors and breast cancer containing tumor-infiltrating mast cells [43]. In the fetal tissues and in the colon mucosa samples, a strong cytoplasmic immunoreactivity for Tβ_4_ was observed in mast cells, paralleling the reactivity for tryptase, in the absence of any reactivity for chymase. In skin samples with dermal-serosal mast cells, Tβ_4_ immunostaining paralleled reactivity of skin-associated mast cells for both chymase and tryptase. A different pattern of expression was found in peri- and intra-tumoral mast cells. In the salivary gland tumors, Tβ_4_ was expressed only in a subset of mast cells, which were immunoreactive for both chymase and tryptase. In breast cancer, most tumor-infiltrating mast cells were reactive for Tβ_4_ and tryptase, whereas immunostaining for chymase was observed in a minority of mast cells. The same pattern of expression was noticed in mast cells surrounding fibrocystic changes in the mammary glands, suggesting that the immunohistochemical pattern observed in breast cancer should not be restricted to cancer, but might reflect the pattern of breast-associated mast cells. All these data extended the knowledge regarding the immunophenotype of mast cells and introduced Tβ_4_ as a new useful marker for the identification of both serosal and mucosal mast cells in normal tissues and in cancer. Even more relevant are the findings regarding the Tβ_4_ expression in intra-tumoral and peri-tumoral mast cells, that might contribute to explain their function in cancer progression. Previous studies have demonstrated the association between high levels of Tβ_4_ and the ability of cancer cells to acquire a motile phenotype, stimulating their directional migration towards lymphatic vessels and capillaries, ending with locoregional and/or distant metastases [44,45,46,47]. In some studies, the expression of Tβ_4_ was not restricted to cancer cells, being observed even in other cell types of the tumor microenvironment (TME) [48]. Moreover, Tβ_4_ was reported to enhance endothelial cell differentiation and angiogenesis, facilitating cancer progression. Our findings on the strong expression of Tβ_4_ in intra-tumoral and peri-tumoral mast cells reinforced the hypothesis that Tβ_4_ should be considered as a new molecular target for antitumor strategies [49]. In addition, since mast cells are one of the multiple constituents of the tumor microenvironment, their ability to produce high levels of Tβ_4_ may suggest the need for developing new anti-cancer strategies focused on the cells of the tumor microenvironment, including mast cells.

## 8. Thymosin β_4_ Cytoplasmic/Nuclear Translocation as a New Marker of Cellular Stress

**Figure 12:**
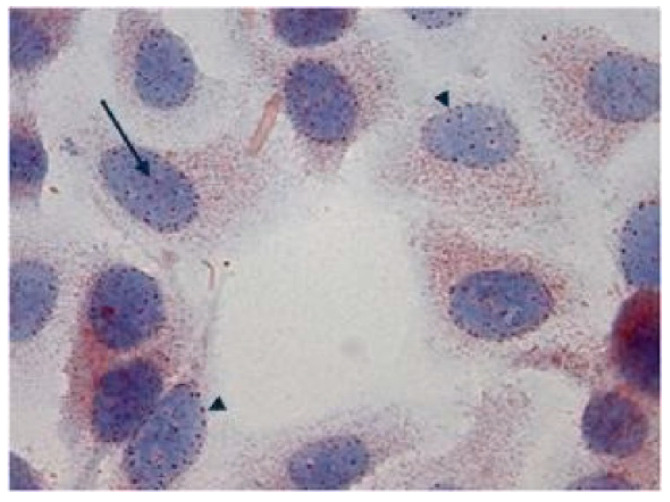
In culture cells under starvation, the appearance of a punctuated pattern of expression in the nuclei is suggestive of the translocation of Tβ_4_ into the nucleus. This pattern of immunoreactivity suggests a preferential localization of the peptide in the nuclear pores. (magnification ×630).

In immunohistochemical studies carried out in fetal and adult tissue samples, Tβ_4_ was always localized in the cytoplasm of cells expressing the peptide, suggesting a prevalent role played by Tβ_4_ in the different cytoplasm compartments, including vesicles (fine granules reactivity), the trans-Golgi network (dots-like deposits in the perinuclear areas), and cytoskeleton-associated actin (homogeneous staining diffused to the entire cytoplasm). Further studies on Tβ_4_ expression in cell culture in different environmental conditions forced us to modify our hypothesis on the restriction of Tβ_4_ expression to the cytoplasm. The first experiment was carried out in HepG2 cells, a line of human hepatocellular carcinoma. In tumor cells growing in normal conditions with fetal bovine serum, in the first 72 h, Tβ_4_ expression was restricted to the cytoplasm, showing a homogeneous reactivity suggestive of the localization of the peptide in the cytoskeleton. At 84 h, the diffused Tβ_4_ cytoplasmic immunostaining shifted to a focal perinuclear reactivity, followed by the appearance of a nuclear immunostaining. In absence of serum, under starvation, nuclear reactivity increased, paralleled by a progressive decrease in cytoplasmic immunostaining. These findings revealed a previously unknown dynamic expression of Tβ_4_, able to translocate from vesicles to the Golgi apparatus and into the nucleus, according to different environmental conditions [49]. The punctuated pattern of associated nuclear Tβ_4_ immunostaining suggested that the peptide might be localized in the nuclear pores (Figure 12), where it could regulate pore permeability.

The normal immunocytochemical pattern was restored when culture cells submitted to starvation for 84 h received a new complete medium for 48 h. This finding evidenced the ability of Tβ_4_ to re-translocate from the nucleus to the cytoplasm, re-acquiring its fundamental function in physiological conditions, mainly related to its actin monomer-sequestering properties with a relevant role in the organization of the cytoskeleton [50]. The translocation of Tβ_4_ from the cytoplasm to the nucleus was confirmed in Caco2 cells, a colon adenocarcinoma cell line [51]. When cells were exposed to multiple stress conditions, including serum starvation and butyrate and DMSO administration, Tβ_4_ translocated from the cytoplasmic domains towards the nucleus. According to these findings, the cytoplasmic/nuclear translocation of Tβ_4_ should be considered a general defensive response against adverse environmental events and indicated Tβ_4_ as an efficient biomarker of early cellular stress [51]. In a more recent experiment on the effects of cellular stress conditions on beta thymosins, cell starvation was found to increase the uptake of extracellular Tβ_4_, increasing the internalization of extracellular Ca^2+^/Tβ_4_ complexes [52]. Given the role of Tβ_4_ and calcium in cell proliferation and migration, the high intracellular concentrations of both Tβ_4_ and calcium ions following starvation might represent the initial signal for the acquisition of a motile phenotype by cancer cells, allowing their detachment from the tumor mass and invasion, ending with the insurgence of the metastatic process.

An immunoelectron microscopic study carried out on HepG2 cells, with the aim of better understanding the intracellular localization of Tβ_4_ in physiological conditions and after starvation, allowed to obtain a better knowledge of the intracellular trafficking of this peptide [53]. In tumor cells growing in a complete medium, Tβ_4_ reactivity was mainly observed in the cytoplasm associated with the endoplasmic reticulum, with a high concentration of gold particles in the perinuclear compartment, often associated with the nuclear membrane. A minor component of Tβ_4_-bound gold particles was also detected inside the nuclear envelope, associated to the nucleolus. Following serum starvation, immunostaining for Tβ_4_ was observed both in the cytoplasm and in the nucleoplasm, whereas only few gold particles decorated the nucleolus. These findings from high-resolution electron microscopy allowed the obtaining of better knowledge regarding the dynamics of intracellular Tβ_4_ trafficking according to different environmental conditions. First, Tβ_4_ was localized in the nucleus even in physiological conditions, being associated with the nucleolus. The low levels of this nucleolar component did not allow its identification in previous immunohistochemical studies. Moreover, new intranuclear trafficking was revealed by the electron immunogold technique: from the nucleolus to the nucleoplasm following serum starvation. Tβ_4_ presence in the nucleolus could reflect the interaction of Tβ_4_ with nucleolar components. According to this hypothesis, Tβ_4_ could contribute, together with other nucleolar acting-binding proteins, to modulating the transcription activity of RNA polymerases.

## 9. Prenatal Thymosin β_4_ Administration Improves Fetal Development

The hypothesis that Tβ_4_ could play a key role in the physiological development of human embryos and fetuses originated from the experimental data emerging from the multiple studies of our group. The initial finding of high concentrations of Tβ_4_ in the saliva of preterm infants was followed by the report of a high expression of the peptide in multiple fetal organs, including salivary glands, gut and annexed glands, kidneys, and the urogenital tract, as described in previous sections of this review. All these observations forced us to better investigate the linkage between Tβ_4_ and human development. Preterm delivery is generally considered one of the most important factors influencing organ development. Preterm labor and low birth weight are associated with the interruption of the development of multiple organs, with a block, after birth, of the genesis of new cardiomyocytes, neurons of the cerebral cortex, and many other cell types, including glomeruli [40]. This block is at the origin of the fetal programing theory, also known as the Barker hypothesis [54]. According to this theory, adverse events occurring during fetal life may influence our susceptibility to develop chronic and acute diseases later in life, in childhood or in adulthood. Infants with a low nephron number are susceptible to renal insufficiency [55]. Newborns with a deficient development of the cardiovascular system should be more susceptible to severe heart attack and/or to severe forms of atherosclerosis [56]; a low number of cortical neurons and glial cells might predispose to the insurgence of neuropsychiatric disorders, including Alzheimer disease and Parkinson disease [57,58,59]. According to the Barker hypothesis, preterm delivery represents an important challenge for the medical community. A question has surfaced in the literature in recent years: how can organ development and nephrogenesis be improved in infants at high risk of undergoing preterm delivery [60,61]? A second question is: is it possible to promote organ development after birth in preterm newborns to transform susceptible infants into individuals resistant to multiple diseases later in life [62]? The hypothesis of “physiological regenerative medicine” [63] has its origin in and its basis on the abundance of stem cells in all the organs of newborns and, particularly, in the organs and tissues of preterm neonates [31]. Stem/progenitor cells represent an optimal target for starting regenerative medicine in the perinatal period [64,65]. Tβ_4_ appeared from our perspective a good candidate for this project, given its multiple physiological roles in actin polymerization, angiogenesis, cell survival, cell migration, and fetal development [66]. Our study was aimed at evaluating the ability of Tβ_4_ to function as a fetal growth promoter when administered to pregnant animals during gestation [67]. To this end, pregnant mice were treated with an intraperitoneal injection of Tβ_4_ on days E14 and E17 of gestation. Maternal Tβ_4_ treatment was associated with a fetal higher cranio-caudal length and with a more accelerated development of lungs, kidneys, heart, and cerebral cortex. Our preliminary data indicated that Tβ_4_ may act as a powerful fetal growth promoter in fetuses when administered to mothers in the prenatal period, transforming the dream of “physiological regenerative medicine” into a possible program to be tested in a large cohort of experimental animals in order to confirm our intriguing preliminary results [68]. However, given that Tβ_4_ is a natural compound, and not a drug, these preliminary data might represent the basis for starting proper clinical trials aimed at introducing the use of Tβ_4_ in clinical practice. The trials might start in pregnant women with a programed preterm delivery and should be focused on this goal: to transform a newborn susceptible of developing chronic diseases later in life into a resistant subject, thanks to the accelerated development of multiple organs operated by the maternal administration of Tβ_4_.

## 10. Concluding Remarks

Several years ago, at an international meeting on β- and α-thymosins organized in Washington by Prof. Goldstein, one of the speakers, Hui Qiao Sun, entitled his presentation “the β-thymosin enigma” [69]. During the presentation, the speaker explained the reasons behind this title, originating from the complex pleiotropic effects of β-thymosins in cells, due to the direct and indirect effects of the cytoskeleton on the modulation of multiple signaling pathways, impacting a variety of cell functions. Over the years, this enigma has not been solved, and the number of functions played by β-thymosins in physiological and pathological conditions has increased, including the influence on angiogenesis, tissue repair, corneal wound healing, collagen deposition, cell survival, stem cell migration, and nephrogenesis [41]. Moreover, they act as anti-apoptotic agents; β-thymosins are involved in fetal development, in wound healing, and in cancer onset and progression. Very recently, Tβ_4_ has been reported to play a key role in the differentiation of thymocytes by influencing cytoskeletal rearrangement and mitochondrial transfer in thymic epithelial cells [70], effects that reinforce the hypothesis of a Tβ_4_ impact on the aggregation of F-actin.

The complexity of immunohistochemical data regarding the expression of Tβ_4_ and Tβ_10_ in different tissues and cell types, often characterized by an uneven and patchy distribution, did not allow a quantitative evaluation of the levels of expression of β-thymosins in different organs and tissues. Further studies based on the application of artificial intelligence, and in particular the use of deep learning models applied to whole-slide images, will probably allow a quantitative determination of the concentrations of these peptides.

In this review, which covers the works of our group for more than 20 years, we described the pleiotropic functions of Tβ_4_ and Tβ_10_ in multiple cell functions during development and in carcinogenesis. New functions of β-thymosins emerge from this review. The expression of β-thymosins in the mesenchymal cells of the tumor microenvironment enlarges the spectrum of key functions played by these peptides during carcinogenesis, functions that are not restricted to tumor cells, including the tumor microenvironment and cancer-associated fibroblasts. Given the role of Tβ_4_ and Tβ_10_ in cancer progression, the Tβ_4_- and Tβ_10_-producing stromal cells of the tumor microenvironment appear an ideal target of future anti-cancer therapies aimed at halting the ability of tumor cells to migrate and create distant metastases through the blocking/inhibition of Tβ_4_ biological action. Therefore, we believe that the results reported in this review contribute to elucidating β-thymosins’ functions in health and disease and offer new clues to solve the “β-thymosin enigma”.
